# Mechanical homeostasis imbalance in hepatic stellate cells activation and hepatic fibrosis

**DOI:** 10.3389/fmolb.2023.1183808

**Published:** 2023-04-20

**Authors:** Yuan-Quan Zhao, Xi-Wen Deng, Guo-Qi Xu, Jie Lin, Hua-Ze Lu, Jie Chen

**Affiliations:** ^1^ Department of Hepatobiliary Surgery, Guangxi Medical University Cancer Hospital, Nanning, China; ^2^ Graduate School of Youjiang Medical University for Nationalities, Baise, China

**Keywords:** hepatic fibrosis, biomechanics, extracellular matrix, hepatic stellate cells, mechanotransduction

## Abstract

Chronic liver disease or repeated damage to hepatocytes can give rise to hepatic fibrosis. Hepatic fibrosis (HF) is a pathological process of excessive sedimentation of extracellular matrix (ECM) proteins such as collagens, glycoproteins, and proteoglycans (PGs) in the hepatic parenchyma. Changes in the composition of the ECM lead to the stiffness of the matrix that destroys its inherent mechanical homeostasis, and a mechanical homeostasis imbalance activates hepatic stellate cells (HSCs) into myofibroblasts, which can overproliferate and secrete large amounts of ECM proteins. Excessive ECM proteins are gradually deposited in the Disse gap, and matrix regeneration fails, which further leads to changes in ECM components and an increase in stiffness, forming a vicious cycle. These processes promote the occurrence and development of hepatic fibrosis. In this review, the dynamic process of ECM remodeling of HF and the activation of HSCs into mechanotransduction signaling pathways for myofibroblasts to participate in HF are discussed. These mechanotransduction signaling pathways may have potential therapeutic targets for repairing or reversing fibrosis.

## 1 Introduction

Mechanical forces can regulate cell function and direct cell behavior, and cells can also maintain homeostasis by responding to mechanical stimuli (Alvarez and Smutny, 2022). Cells sense mechanical forces generated by the extracellular matrix (ECM) and convert them into biochemical signals that are transmitted into the nucleus, causing specific cellular responses (such as regulating gene transcription or altering the cytoskeleton), a process known as mechanotransduction (Kang, 2020). Hepatic fibrosis (HF) can be caused by a variety of causes, including autoimmune hepatitis, viral hepatitis, non-alcoholic fatty liver disease, and alcoholic liver disease, among others (Berumen et al., 2021). It is noteworthy that HF is a pathological process of liver cirrhosis and hepatocellular carcinoma (HCC), and its main complications include portal hypertension and liver failure (Parola and Pinzani, 2019). HF is essentially the result of abnormal repair of liver tissue, characterized by excessive deposition of ECM. The ECM of the fibrotic liver is mainly produced by myofibroblasts, and hepatic MFBs are mainly derived from activated hepatic stellate cells (HSCs) (Kovner et al., 2022). Under physiological conditions, HSCs are silent in the liver and are activated to participate in HF after liver injury. Activated HSCs can secrete a variety of cytokines, induce ECM (such as collagens) to increase, regulate matrix metalloproteinases (MMPs) and tissue inhibitors of metalloproteinases (TIMPs), and destroy ECM homeostasis. When the liver injury is repaired, HSCs are deactivated or apoptosis and fibrosis are reversed (Caligiuri et al., 2021). In recent years, many studies have found that HSCs can sense the mechanical force generated by ECM and convert it into biochemical signals to participate in the occurrence and development of HF. This review will focus on the mechanotransduction signaling pathways of HSCs activation and the mechanism of mechanical homeostasis imbalance in HF.

## 2 Introduction to the extracellular matrix

In most organs of the human body, the ECM is mainly composed of the interstitial matrix and the basement membrane, the latter comprising the epithelial cell basement membrane and the endothelial cell basement membrane (Burgstaller et al., 2017). The ECM is a macromolecular network composed of collagens, elastin, fibronectin, laminins, glycosaminoglycans (GAGs), proteoglycans (PGs), and glycoproteins. It not only provides mechanical elastic structural support for organ cells but also regulates cell proliferation, survival, migration, differentiation, and morphological changes (Theocharis et al., 2016; Li et al., 2021). Collagens are important components of mechanical homeostasis, providing structural support for connective tissue and forming the basement membrane and interstitial matrix of organs (Badylak et al., 2015; Cramer and Badylak, 2020). There are many types of collagen, among which type I, type II, type III, type IV, type V, and type VI are more common (Mienaltowski et al., 2021). The most abundant collagens in the liver are proto-fibrous collagens (I, III, V) in the interstitial matrix, while proto-microfibrous collagen type VI and basement membrane collagens (IV, XVIII) are less abundant in the liver (Karsdal et al., 2017). Type IV collagen, which together with laminin constitutes the major component of the basement membrane of epithelial and endothelial cells, can be assembled into a loose permeable network that allows the bidirectional flow of nutrients and metabolites from the blood to the hepatocytes (Leeming et al., 2012). Collagens (I, II, III, V, XI) belong to proto-fibrous collagens, which are mainly involved in the composition of interstitial matrix, forming a dense diffusion barrier network, which is the core of tissue structure and integrity (Karsdal et al., 2017). This is a kind of supporting scaffold, which can be produced by different types of fibroblasts. In biomechanics, type I collagen is the main material that changes the mechanical properties of tissue and ECM, and its content is positively correlated with organ stiffness (Swift et al., 2013; Swift and Discher, 2014). Elastin, fibronectin, laminins, GAGs, and PGs belong to the non-collagenous protein family in ECM. Elastin is present in the walls of blood vessels, the capsule, and long-term fibrotic tissues, and can provide reversible elasticity to the organ to relieve mechanical loading (Cocciolone et al., 2018). GAGs are fibrous, rigid, unbranched polysaccharide chains that are composed of repeating disaccharide units, present as free sugar chains [e.g., hyaluronic acid (HA) ], or attached primarily to PGs (e.g., heparan sulfate or chondroitin sulfate chains) (Purushothaman et al., 2023). PGs are hydrated gels with a certain consistency that regulate cell adhesion and maintain hyperosmolality (Khurana et al., 2021). Fibronectin exists in the liver capsule, portal vein interstitium, and septal space, which is closely related to liver development and hepatocyte survival after acute liver injury. Fibronectin can promote the interaction between heparins and collagens, mediate cell adhesion, and participate in many biological processes, such as coagulation, wound healing, and ECM assembly (Miron-Mendoza et al., 2017; Jara et al., 2020). Compared with acute liver injury, HF persists as a wound repair response in chronic liver injury, and fibrosis continues to thicken, impairing tissue structure and function. After the liver injury, the low-density basement membrane-like matrix in the Disse space is destroyed, and the original collagen types IV and VI are replaced by collagen types I, III, and fibrin (Hernandez-Gea and Friedman, 2011). Laminins exist in the basement membrane and endoplasmic reticulum of mesothelial cells, which can affect cell adhesion and movement, regulate cell growth and differentiation, and participate in the progress of fibrosis, tumor invasion, and metastasis. Integrin families belong to a class of glycoproteins in ECM, which can act as ligands of cell surface receptors, regulate cell proliferation, migration, differentiation, and wound healing, and participate in a wide range of biological processes, including ECM assembly and cell-ECM interaction (Hudson et al., 2017; Horton, 2021).

In addition, ECM also contains MMPs, TIMPs, lysyl oxidases (LOX), and lysyl oxidase-like enzymes (LOXLs) and a series of growth factors, which play an important role in the formation and regression of fibrosis. MMPs are zinc-dependent endopeptidases, which can degrade all components of ECM and a large number of non-matrix proteins, and participate in regulating the activities of pro-inflammatory factors, growth factors, chemokines, and so on (Laronha and Caldeira, 2020). TIMPs are natural antagonists of MMPs, which can reversibly inhibit activated MMPs and cooperate with MMPs to regulate the balance between ECM formation and hydrolysis in the liver (Geervliet and Bansal, 2020; Shan et al., 2023). LOX and LOXLs can promote the crosslinking of collagens and elastin, contribute to ECM fibrosis, and delay the reversal of HF induced by carbon tetrachloride (Liu et al., 2016). Therefore, when the synthesis and degradation of liver ECM are unbalanced, homeostasis is destroyed, which will lead to the destruction of liver tissue structure and cause diseases (such as fibrosis and cancer).

## 3 Extracellular matrix remodeling in hepatic fibrosis

The progression of chronic liver disease (CLD), including alcoholic liver disease (ALD), non-alcoholic fatty liver disease (NAFLD), non-alcoholic steatohepatitis (NASH), and hepatitis B and C viral (HBV/HCV), is associated with the development of HF. HF formation is a continuous process and can be classified as portal fibrosis (F1), moderate fibrosis (F2), severe fibrosis (F3), and cirrhosis (F4) according to the Metavir score. It is well known that the main feature of HF is that persistent liver injury leads to excessive deposition of ECM proteins, which mainly include fibrous proteins (such as collagens, fibronectin, elastin, and laminins) and GAGs (such as HA and heparin sulfate). In the process of fibrosis formation, the deposition of ECM proteins changes the content and composition of ECM components. For example, compared with a healthy liver, the number of ECM proteins in the fibrotic liver increases by 6 times (Roehlen et al., 2020), and the number of ECM proteins in the cirrhotic liver can be as high as 10 times (Karsdal et al., 2017), which will lead to changes in ECM biochemical and mechanical properties. In addition, ECM protein deposition triggers the remodeling of liver tissue, leading to extensive fibrous scarring, distortion of liver structure, and significant impairment of liver function (Khanam et al., 2021). We call the process of ECM deposition during fibrosis resulting in changes in its component content and composition ECM remodeling, which is a complex and highly coordinated process with its synthesis, secretion, degradation, and recombination constantly changing dynamically with the progression of fibrosis. In essence, it is a repair reaction of tissues and organs, and a protection mechanism activated in response to stress and injury to maintain the functional and anatomical integrity of the liver.

As one of the main components of ECM structure, almost all types of collagen are increased during ECM remodeling in HF. For example, the synthesis and deposition of type I collagen in the fibrotic liver can be 8 times higher than in the healthy liver (Crawford and Burt, 2012). In an earlier study of dimethylnitrosamine-induced HF in rats (Ala-Kokko et al., 1987), the mRNA concentrations of type I, III, and IV collagen increased in the early stage of HF, but the increase of type IV collagen was particularly significant in the early stage of fibrosis, and the accumulation of type I and III collagen was mainly concentrated in the late stage of fibrosis. In a study of CCl 4-induced HF in mice, type I collagen expression was significantly elevated in the early stage of fibrosis and continued to increase in the late stage of fibrosis, while elastin synthesis and accumulation increased only in the late stage of fibrosis (Chen et al., 2019). In a study of ALD patients, compared with matched healthy patients, ALD patients had higher levels of collagens (III, IV, V, and VI) formation and degradation, and with the progression of fibrosis, collagens (III, IV, and V) formation was relatively more than degradation, except for collagen VI, and this imbalance of ECM remodeling was particularly evident in interstitial matrix type III collagen (Thiele et al., 2021). A liver biopsy study (Baiocchini et al., 2016) of 57 patients with HF according to Metavir classification used a decellularized method to purify ECM scaffolds from human liver tissue to dynamically understand the process of ECM remodeling. The results of this study show that the evolution of HF from F1 to F3 requires a gradual accumulation of collagens (I, III) and elastin, but the achievement of cirrhosis (F4) involves a large accumulation of elastic fibers (i.e., overexpression of elastin), accompanied by a modest reduction of type I and III collagen. Thus, since each collagen has a different role in the extracellular matrix of the liver, it is likely that their increased amounts are not the same at the same stage of HF. There are many kinds of collagens in ECM, and they show different increasing patterns in the process of fibrosis. The expression of some collagens is dominant in the early stage of HF, while the expression of other collagens may be more prominent in the late stage of fibrosis. In addition, there are many causes of HF, and the ECM remodeling process of HF may also vary from different etiologies. To date, the detailed process of ECM remodeling in HF and the characteristics of ECM remodeling in different etiologies of HF are still unclear and need to be further confirmed.

GAGs and PGs, as another major molecular type of ECM, are also involved in extracellular matrix remodeling in HF. GAGs are linear polyanionic animal heteropolysaccharides, which can be classified into four groups according to the structure of disaccharide units: heparin/heparin sulfate, chondroitin/chondroitin sulfate, keratin sulfate, and HA (Song Y. et al., 2021). PGs are a class of modified proteins formed by the covalent binding of GAGs (such as chondroitin sulfate or heparin sulfate chains) other than HA to the protein core (Wang and Chi, 2022; Purushothaman et al., 2023). PGs have been shown to enhance the remodeling capacity of liver tissue, and their numbers are significantly increased in cirrhotic liver tumors, especially perlecan and decorin, and the knockout of perlecan and decorin also leads to a decrease in ECM stiffness (Kovalszky et al., 1993; Kimura et al., 2008; Robinson et al., 2017). Syndecans are a family of four-member transmembrane proteoglycans that primarily carry heparan sulfate chains, including Syndecan-1(SDC-1), Syndecan-2(SDC-2), Syndecan-3(SDC-3), Syndecan-4(SDC-4). During the transformation of HSCs into myofibroblasts, a key process in the development of cirrhosis, the mRNA levels of syndecan-1, -3, and -4 remained unchanged, whereas the mRNA amount of syndecan-2 increased (Weiner et al., 1996). SDC-1 is overexpressed in the early stages of HF and may inhibit fibrosis by interfering with TGFβ1 and by promoting the upregulation of matrix metalloproteinase 14, a protease that plays a key role in matrix degradation (Regős et al., 2018). Notably, this protective effect of SDC-1 on HF may change over time. Further studies confirmed that the overexpression of SDC-1 was positively correlated with the degree of HF, and the expression of SDC-1 was significantly increased in advanced HF compared with early and middle HF (Charchanti et al., 2021). Agrin is a heparan sulfate proteoglycan that is expressed or secreted into the ECM as a membrane proteoglycan (Mead et al., 2022) and is aggregated in chemically induced cirrhotic and hepatoma rat livers (Tátrai et al., 2006). Furthermore, during HF and liver cancer development, excessive accumulation of agrin, in addition to collagens, laminins, and elastin, will lead to the formation of a stiffer ECM (Chang et al., 2017). HA, the only unsulfated GAGs, is generally considered to be a biomarker elevated in the blood of patients with HF and cirrhosis (Matsuda and Seki, 2020b; Kim and Seki, 2023), and is actively synthesized during the HF process, its accumulation starting in early HF (F1-2) and significantly increasing in F3-F4 fibrosis (Kim and Seki, 2023).

Another feature of HF is that the stiffness of the ECM can be increased due to the large deposition of ECM proteins, and the increased stiffness of the ECM can affect cellular biological behavior through mechanotransduction (Lachowski et al., 2019). ECM stiffness refers to the ability of ECM to resist elastic deformation when subjected to force. Matrix stiffness contributes to the mechanical properties of ECM proteins and can affect the behavior of HSCs, including growth, movement, adhesion, and differentiation into myofibroblasts (Wells, 2008). One of the important aspects related to matrix stiffness in the process of HF is the imbalance of major enzymes involved in ECM degradation, such as MMPs and their inhibitors TIMPs. MMPs are key mediators of ECM remodeling, and their expression changes during fibrosis progression, such as MMP-1 decreases in fibrotic livers, while MMP-2 increases with fibrosis progression (Iredale et al., 2013). Similarly, the expression of TIMP-1 and TIMP-2 significantly increases in fibrosis (Leroy et al., 2004). In the process of ECM remodeling, TIMPs inhibit the activity of MMPs, and the synthesis and degradation of ECM are unbalanced, resulting in a net increase in ECM deposition. An *in vivo* study in mice found that lysyl oxidase-like 1 (Loxl1) expression was elevated only in the late stages of HF, and inhibition of Loxl1 expression significantly reduced elastin crosslinking, thereby preventing HF progression (Zhao et al., 2018). The simultaneous increase in the expression of tropoelastin (a precursor molecule of elastin) and Loxl1 may lead to the cross-linking and subsequent accumulation of elastin in advanced HF, and by the time of cirrhosis, the overaccumulation of elastin may bind to the collagen scaffold, thereby promoting collagen fibers into a more contractile state and leading to increased HF(Chen et al., 2019). Studies have shown that fibrosis is associated with increased matrix stiffness due to excessive collagen deposition and cross-linking (Wells, 2013). Therefore, the progression of HF may alter liver stiffness through the binding of collagen and elastic fibers, and the increased matrix stiffness caused by the net accumulation of elastin may be responsible for the progression of advanced fibrosis to cirrhosis.

In general, there are many causes of HF, and the process of ECM remodeling may be different for different causes of HF. Up to now, the detailed process of ECM remodeling in HF and the characteristics of ECM remodeling in different etiological backgrounds are not clear, and further studies are expected to confirm this.

## 4 The effects of biomechanics on hepatic stellate cell activation

HSCs are a kind of non-parenchymal cells peculiar to the liver, which are the main cell group for the synthesis of ECM in the liver and distribute in the Disse space between liver sinusoidal endothelial cells (LSECs) and hepatocytes. Under normal physiological conditions, HSCs are resting, storing vitamin A in the form of retinyl esters in their nuclear droplets, also known as hepatic fat-storing cells (Cai et al., 2020). When the liver is stimulated by mechanical signals or inflammation, HSCs will proliferate and activate into myofibroblasts and express α-smooth muscle actin (α-SMA) and type I collagen in large quantities, which promotes fibrosis and wound healing. When the injury and inflammation subside, myofibroblasts decrease (such as apoptosis or return to static phenotype) (Trivedi et al., 2021). Activated HSCs can continuously proliferate and secrete a large amount of ECM proteins in the liver. Under normal circumstances, matrix production and degradation are in a dynamic balance, and once the amount of matrix production exceeds the protease degradation capacity, the wound repair response promotes HF. The differences between myofibroblasts versus quiescent HSCs are shown in Table 1.

**TABLE 1:** The differences between myofibroblasts versus quiescent HSCs that cause ECM imbalance and hepatic fibrosis.

Quiescent HSCs	Myofibroblasts
Cell bodies ovoid or irregularly shaped and often projecting several stellate projections	Cell bodies are larger and the stellate projections are stretched and thinned
Rich in retinoid droplets	Retinoid loss
ECM homeostasis	α-SMA and collagen-I abundant expression
Non-proliferative	Proliferation
Expression of small amounts of TGF-β, PDGF, IGF and other cytokines	Accumulation of ECM proteins
	Contractility and chemotaxis
	Increased cross-linking and promoting Fibrogenesis
	Altered matrix degradation
	Involved in inflflammatory and mechanical signaling

In addition to the activation of HSCs by changes in ECM components, transforming growth factor-β1 (TGF-β1) is also involved in the activation process and plays a key role (Dooley and ten Dijke, 2012). TGF-β1 is stored in the ECM and responds to perturbations in the microenvironment to ensure ECM homeostasis. Secreted TGF-β1 remains inactive by binding to a protein complex called the large latency complexes (LLCs). Instead, LLCs are anchored on fibril ECM proteins (fibronectin, fibrillin, fibrin) by potential TGF-β-binding proteins (LTBP proteins) and form an inactive TGF-β1 repository within the ECM (Hinz, 2015). Activated TGF-β1 is released from LLC by proteolysis or mechanical deformation of the complex. Integrin-carrying cells bind to latency-associated protein (LAP) in the ECM-anchored LLC, which creates a pulling force that results in a conformational change in the LLC that releases activated TGF-β1 (Shi et al., 2011; Murphy-Ullrich and Suto, 2018). When the mechanical resistance of the ECM is guaranteed to be applied to the LAP, it will produce the conformational changes required for releasing TGF-β 1. The disorganized ECM (typically remodeled tissue) does not provide sufficient resistance to induce TGF-β1 release, while the stiff ECM (typically advanced fibrosis) is expected to ensure adequate resistance to latent TGF-β1 activation by cell traction (Hinz, 2015). During the formation of hepatic fibrosis, excessive TGF-β1 can activate HSCs and induce their differentiation into myofibroblasts, further leading to ECM protein deposition and ECM stiffness. Similarly, the timely release of activated TGF-β1 can further drive proinflammatory and inhibitory immune responses in response to changes in ECM stiffness during the progression of fibrosis (McQuitty et al., 2020). The integrin-dependent mechanical release of TGF-β1 is closely related to ECM stiffness, and increasing ECM stiffness has a direct impact on the activation of TGF-β1, thereby regulating the activation of HSCs.

**FIGURE 1:**
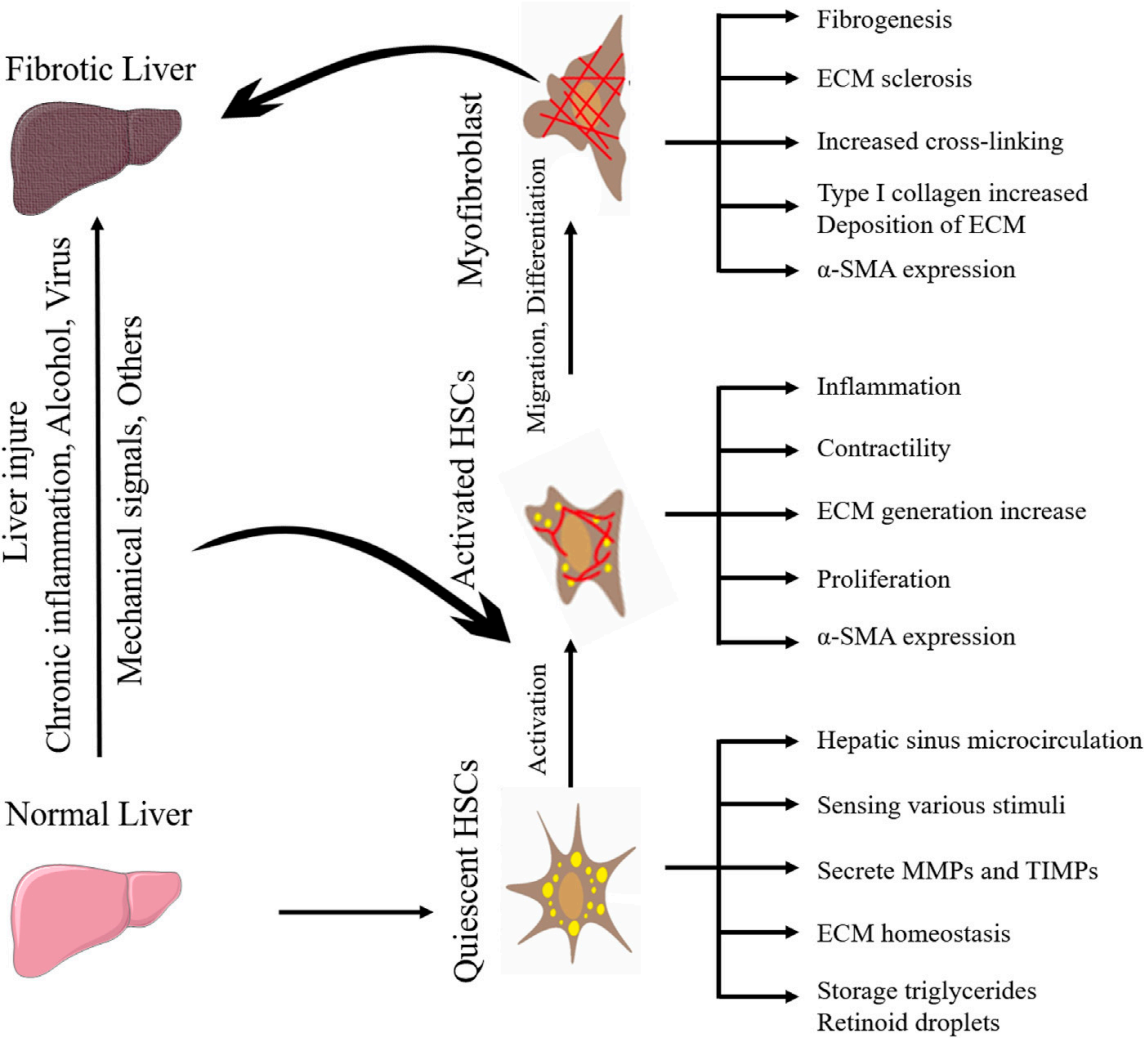
The activation process of quiescent HSCs into myofibroblasts during hepatic fibrosis.

Moreover, HSCs can migrate and aggregate to stiff areas through mechanotransduction pathways (described below), further stiffening the extracellular environment (Olsen et al., 2011; Kang, 2020). Therefore, the activation of HSCs is the central event in HF and the key entry point for preventing or reversing HF. Understanding the mechanism between the changes in ECM components and the activation of HSCs is the key to finding potential therapeutic targets for HF. The activation process of quiescent HSCs into myofibroblasts during HF is shown in Figure 1. The perception, transformation, and conduction of mechanical forces generated by the stiff extracellular environment by intrahepatic cells as well as their effect on target cells is a complex process. When the mechanical force of the ECM is sensed by mechanoreceptors on the plasma membrane, it is amplified by the protein complex, converted into biochemical signals, and transmitted downward; finally, through the connection between the cytoskeleton and the nucleoskeleton, the signal is transmitted to the nucleus to change gene transcription in response to mechanical forces (Kang, 2020). Integrins at ECM junctions, cadherins, cell adhesion molecules at cell-cell contacts, mechanically sensitive ion channels, and tyrosine kinase receptors are mechanoreceptor receptors on the hepatic cytoplasmic membrane (Lim et al., 2018). ECM stiffness can be detected by HSCs surface receptors, such as integrins, leading to HSCs activation (Caliari et al., 2016). ECM composition changes and density and stiffness increases can act as mechanical stimulation, at least in part by activating HSCs through the integrin signaling pathway to form a positive feedback loop (Higashi et al., 2017). HSCs are mechanically transduced in a stiff environment to promote activation and secretion of matrix components, further stiffening the cellular environment, and forming a positive feedback loop, so the stiffened ECM is not only the cause but also the result of HSCs activation.

The liver is an immune organ in the human body that contains multiple immune cell types from the innate and adaptive immune systems. HSCs can interact with a variety of cells in the liver due to their special anatomical location in the Disse space. Similarly, HSCs can express multiple receptors and soluble effector molecules, allowing them to interact with different subsets of immune cells in the liver. The immune cell subsets contained in the liver include macrophages, dendritic cells (DC), natural killer cells (NK), and natural killer T cells (NKT), etc. Studies have pointed out that macrophages may sense extracellular mechanical stimulation by activating and recruiting into the liver, thus secreting specific cytokines and chemokines to activate HSCs and participate in the progression of HF. Macrophages in the liver can be divided into resident macrophages, Kupffer cells, and macrophages derived from bone marrow monocytes. In the event of liver injury, Kupffer cells have the sentinel function of phagocytosing harmful environmental substances and regulating the liver immune response, while monocyte-derived macrophages are cells that produce inflammatory cytokines, which help to timely regulate the liver inflammatory response and wound healing response (Matsuda and Seki, 2020a). In addition, when liver damage or inflammation occurs, macrophages can differentiate into M1-type macrophages (proinflammatory effect) of the classic activation pathway or M2-type macrophages (anti-inflammatory, tissue repair, and remodeling action) of alternating activation pathways, express corresponding molecular markers and regulate liver inflammation and fibrosis (Wan et al., 2014). Macrophages can also interact with HSCs, leading to the remodeling of the immune microenvironment and ECM (Cheng et al., 2021). From the perspective of body immunity, HF is a wound healing response regulated by the immune system, and mechanical forces can continuously activate the acute inflammatory pathway, thus forming an environment similar to chronic inflammation, and the prolonged inflammatory period leads to fibrosis. *In vitro* experiments have shown that the effect of mechanical forces on macrophage phenotype is bidirectional, and the application of circulatory pull to macrophages adhering to the scaffold can observe that the M2/MI ratio increases at 7% strain and decreases at 12% strain, suggesting that lower tension may maintain microenvironmental homeostasis, while abnormal stretching may initiate an inflammatory response (Ballotta et al., 2014). One study (Gruber et al., 2018) has shown that macrophages can sense ECM stiffness through mechanotransduction and respond by modulating Toll-like receptor (TLR)-mediated inflammatory responses. For example, macrophages grown on softer substrates induce stronger TLR4 and TLR9 signaling and release the more potent proinflammatory cytokine TNF-α. There is evidence that several physical factors of the ECM influence macrophage polarization and determine the outcome of wound healing and tissue remodeling, including stiffness and topography (Soliman et al., 2011; Qiu et al., 2018). A recent experiment (Chen et al., 2020) showed that substrate stiffness modulates bone marrow-derived macrophage (BMM) polarization through the nuclear factor κB (NF-κB) signaling pathway, with low substrate stiffness promoting BMM transfer to classically activated macrophages (M1) by modulating the NF-κB pathway initiated by reactive oxygen species (ROS) and medium substrate stiffness promoting BMM transfer to alternately activated macrophages (M2). Therefore, mechanical stimuli (such as changes in microenvironmental mechanical forces) generated during ECM remodeling and stiffening in HF may be sensed by macrophages through mechanotransduction pathways and initiate hepatic inflammatory responses through specific receptors. Then, liver inflammation can further trigger the activation of liver macrophages and their recruitment to the liver to produce cytokines and chemokines, including transforming growth factor β (TGF-β), platelet-derived growth factor (PDGF), and tumor necrosis factor (TNF), to induce the activation of HSCs, thereby regulating HF (Tsuchida and Friedman, 2017). TGF-β is mainly derived from activated macrophages, which are highly related to the promotion of HF. In the promotion of HF, TGF-β can enhance the destruction of hepatocytes, mediate the activation of HSCs and fibroblasts, and cause ECM deposition. For example, it can promote the production of type I and type III collagen in HSCs through a Smad-dependent pathway (Dooley and ten Dijke, 2012). TGF-β can also directly affect collagen production changes in hepatic stellate cells through integrins and promote the production of type I and III collagen, and in a mouse model of integrin αvβ8 knockout, fibroblasts showed decreased reactivity to TGF-β and decreased proliferative ability (Walton et al., 2017). The PDGF produced by activated macrophages can promote the process of HF by promoting the proliferation and migration of HSCs, activating HSCs through the PDGF-B, PDGF-C, PDGF-D/PDGFR-β pathways, and further strengthening the fibrosis response through the extracellular regulated protein kinase (ERK), AKT (a serine/threonine kinase), and NF-κB pathways (Bonnardel et al., 2019). Tumor necrosis factor (TNF) is a cytokine that can be divided into TNF-α and TNF-β, of which TNF-α, although not directly inducing type I collagen production in HSCs, can promote fibrosis by upregulating TIMP-1, downregulating bone morphogenetic protein and activin membrane-bound inhibitor (BAMBI), and preventing HSC apoptosis (Tarrats et al., 2011; Osawa et al., 2013; Liu et al., 2014). Therefore, hepatic macrophages may sense ECM stiffening through mechanotransduction and polarize into M1 or M2, produce corresponding cytokines and chemokines, and regulate HSCs through intercellular communication, thereby participating in the progression and regression of HF. This also indicates that there is interaction and crosstalk between mechanical transduction and biochemical signal transduction in the process of HF. However, the specific mechanism by which hepatic macrophages perceive mechanical stimulation to polarize is unclear, little research has been done, and the correlation between biomechanical signals and the activated form of hepatic macrophages (M1 or M2) is poorly understood.

## 5 Mechanotransduction in hepatic fibrosis

Currently, biomechanics have been extensively studied in the lung, kidneys, and liver. Mechanotransduction refers to the process by which cells activate cellular pathways and affect their functions by converting mechanical stimuli generated by the extracellular environment into biochemical signals (Deng et al., 2020). Intrahepatic cells can sense changes in matrix mechanics and make changes to this through mechanotransduction to adapt to the changing environment, in which ECM proteins, especially collagens, give signals to the correct position, migration, activation, and metabolic capacity of cells attached to them. Integrin is a specific ECM receptor, and the external force applied to the cell can transfer mechanical forces from the ECM to the cytoskeleton through integrin-mediated adhesions (Lim et al., 2018) and can also cause the aggregation of integrins, thereby promoting actin polymerization, followed by enhanced adhesion stability and contractility, and finally activating downstream transcriptional effectors in the mechanical signaling pathway to achieve HF regulation (Sun et al., 2016). The mechanotransduction pathways that have been identified or may be potential in HF are described below.

### 5.1 Hippo-YAP/TAZ signaling pathway

Yes-associated protein (YAP) and TAZ (WWTR1, a transcriptional activator with a PDZ-binding motif) are downstream molecules of the Hippo pathway, which are involved in liver development, regeneration, and injury repair (Pibiri and Simbula, 2022). *In vivo* experiments in mice, the experimental activation of YAP/TAZ promoted the regeneration of damaged organs, and overactivation led to liver fibroblasts entering a fibrotic state, which intensified the inflammatory fibrosis of the liver and promoted the occurrence of liver tumors, leading to poor prognosis (Moya and Halder, 2019; Mooring et al., 2020; Yeh et al., 2021). YAP/TAZ is a mechanosensor and mediator that can sense mechanical cues arising from changes in ECM stiffness, cell geometry, cell density, and actin cytoskeletal state. The Hippo-YAP/TAZ signaling pathway can be activated by these mechanical signals and is also regulated by cell adhesion and cell polarity (Moon et al., 2019).

The Hippo-YAP/TAZ signaling pathway consists of a kinase cascade, mammalian sterile line 20-like kinase 1 (MST1, also known as STK4) and mammalian sterile line 20-like kinase 2 (MST2, also known as STK3), large tumor suppressor protein 1 (LATS1) and large tumor suppressor protein 2 (LATS2), linker proteins SAV1 (SAV1), MOB kinase activators 1A (MOB1A) and MOB kinase activators 1B (MOB1B), and YAP/TAZ. MST1/2 plays a central role in the regulation of the activity of YAP and TAZ proteins. The MST1/2 kinase cascade can phosphorylate YAP and TAZ proteins to inactivate them and cannot interact with TEAD transcription factors to activate their target gene transcription (Mo et al., 2012). The cell-ECM adhesion site integrin complex mediates mechanical signals from the ECM to the Hippo pathway, which is closely related to actomyosin cytoskeleton changes, and the integrity and polymerization of F-actin can affect cell morphology and cell proliferation (Aragona et al., 2013; Russell and Camargo, 2022). The regulation of the F-actin level has a significant effect on YAP/TAZ activity, which is achieved through a Hippo pathway (LATS1/2 kinase) that is dependent and independent, and F-actin inhibition can activate LATS1/2 and finally lead to phosphorylation inactivation of the YAP protein (Yeh et al., 2021).

In addition, YAP/TAZ is also regulated by focal adhesions (FAs), but the signal from it inhibits the Hippo pathway, to activate YAP/TAZ protein and lead YAP/TAZ to enter the nucleus of HSCs to increase the expression of target genes so that cells can proliferate and secrete ECM to adapt to the stiffened external environment (Meng et al., 2018).

### 5.2 Rho-ROCK signaling pathway

Rho-ROCK is a signaling pathway upstream of Hippo. Rho guanosine triphosphatases (Rho GTPases), belonging to the Ras family, are small signal G proteins that can regulate actin dynamics and actin polymerization by regulating a variety of downstream effector proteins, including ROCK proteins, and they play different functions according to cell types, such as actin-cytoskeleton assembly, cell contraction, and stress fiber formation (Julian and Olson, 2014). In addition, Rho/ROCK regulation of actin cytoskeletal dynamics is related to the nuclear localization and activity of two sets of transcriptional coactivators, myocardin-related transcription factors (MRTFs) and YAP/TAZ (Mason et al., 2019; Yang and Shi, 2021).

The cytoskeleton is an important means of mechanical force transmission in cells, and Rho directs morphological changes associated with HSCs activation by regulating the actin cytoskeleton (Cui et al., 2014). Stress fibers and Fas are the major components of the actin cytoskeleton, and stress fibers are bundles of F-actin with one end inserted into Fas and the other end inserted into a fixed perinuclear or other Fas (Ridley and Hall, 1992). RhoA is a member of the Ras homologous family, which is highly expressed in HF tissues (Wan et al., 2020). Mechanical forces in the ECM activate the RhoA/ROCK mechanical signaling pathway through mechanical sensors such as integrin, and then ROCK induces the phosphorylation of myosin light chain (MLC), increases the expression of transforming growth factor-β (TGF-β) and α-SMA proteins, and activates HSCs to promote HF(Parsons et al., 2010).

In an *in vitro* experiment, it was found that 50 mmHg hydrostatic pressure can significantly induce HSCs to acquire profibrotic properties through the RhoA/ROCK signaling pathway, and ROCK1 mediates the activation of this pathway (Huang et al., 2022). In an earlier experiment (Tada et al., 2001), Y27632, as a Rho kinase inhibitor, was able to partially block the activation of HSCs to inhibit HF *in vitro* and *in vivo*, which was manifested by a significant decrease in α-SMA protein expression and a significant inhibition of type I collagen mRNA transcription. A recent trial showed that both Y-27632 and Y-33075, another Rho kinase inhibitor, significantly reduced contraction, fibrosis, and proliferation of activated primary mouse and human HSCs, but increased HSCs migration (Bachtler et al., 2023). These results suggest that mechanical force can induce the activation of HSCs through RhoA/ROCK signaling pathway and promote the development of HF. Inhibition of this signaling pathway may block or weaken the activation of HSCs, thus hindering the development of HF.

### 5.3 MRTF-A signaling pathway

Myocardin-related transcription factors (MRTFs) are mechanosensory that link actin dynamics, including MRTF-A and MRTF-B, the former interacting primarily with serum response factor (SRF) in the nucleus and subsequently activating target gene transcription (Konopa et al., 2022). MRTF-A (also known as MKL-1) binds to G-actin and is sequestered in the cytoplasm, then shuttles from the cytoplasm to the nucleus, which is controlled by actin polymerization mediated by the RhoA-ROCK pathway (Rahman et al., 2018; Solagna et al., 2020). When RhoA binds to the downstream effector molecule ROCK, G-actin polymerizes into F-actin, and MRTF-A dissociates from G-actin, triggering MRTF-A translocation to the nucleus, interacting with SRF and forming an active SRF/MRTF-A complex, which then activates target genes containing CArG elements, resulting in the expression of a large number of fibrotic proteins and extracellular matrix components, such as α-SMA gene expression and type I collagen synthesis (Shi and Rockey, 2017; Montagner and Dupont, 2020; Al-Hetty et al., 2022). Kong et al. found that SRF deficiency slowed the activation of HSCs *in vitro*, and that conditional SRF deficiency in HSCs attenuated bile duct ligation (BDL) and carbon tetrachloride-induced HF in mice (Kong et al., 2019). CCG-1423 is a new small molecule inhibitor of the MRTF-A mechanism that can block the transcription of MRTF-A by functionally inhibiting its nuclear localization, and it has been found in experiments that CCG-1423 has a good effect in the treatment of HF and not only blocks the expression of MRTF-A and α-SMA protein but also reduces the degree of HF and collagen content (Hayashi et al., 2014; Zheng et al., 2017). Shi et al. found that CCG-203971 (another novel small molecule inhibitor) can block the nuclear translocation pathway of cardiac muscle and MRTF-A, directly inhibit the activation of Smad 2/3 in SRF and type I collagen α2 promoter mediated by cardiac muscle/MRTF-A, and indirectly abolish the actin cytoskeleton-dependent regulation of Smad 2/3 and Erk 1/2 phosphorylation and nuclear accumulation, and finally lead to dose-dependent inhibition of actin cytoskeleton dynamics in HSCs.and abrogated multiple functional features of HSCs activation (i.e., HSCs contraction, migration, and proliferation), as well as decreased expression of type I collagen *in vitro* and HF *in vivo* (Shi et al., 2020). These results suggest that the interaction between MRTF-A translocation and SRF is the key to the activation of target genes of the MRTF-A signaling pathway, and the prevention of G-actin cytoskeleton destruction is crucial for MRTF-A translocation into the nucleus, and the input of actin mechanical signal is the premise of pathway triggering. In the future, the MRTF-A signaling pathway may become a new approach for the targeted treatment of HF.

**FIGURE 2:**
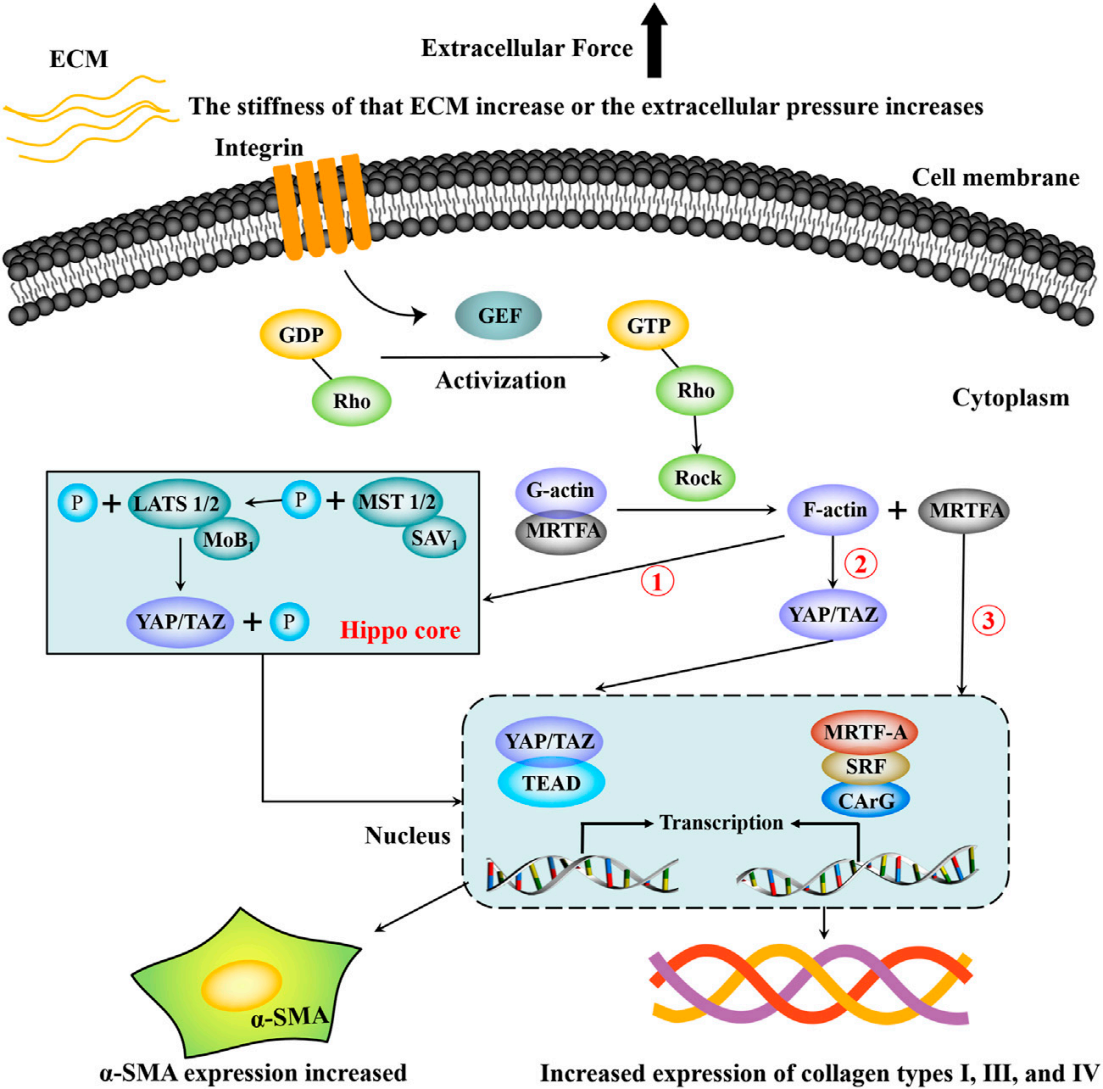
Partial mechanotransduction pathways in the process of hepatic fibrosis. Integrin senses mechanical signals such as extracellular matrix pressure, which can increase the guanylate conversion factor (GEF), and GEF promotes the phosphorylation of GDP-Rho to GTP-Rho, which promotes increased ROCK. Under the continuous action of ROCK, the complex composed of G-actin and MRTF-A dissociates, G-actin is converted into F-actin and re-rehearsed, and F-actin can directly activate YAP/TAZ to translocation into the nucleus. F-actin can also inhibit the phosphorylation of MST1/2, thereby blocking the phosphorylation of LATS1/2, resulting in the inability of YAP/TAZ to be phosphorylated and degraded. Then YAP/TAZ accumulated in the cytoplasm enters the nucleus and binds to TEAP, increasing the transcriptional expression of target genes. In addition, dissociated MRTF-A enters the nucleus and binds to serum response factor (SRF) and then activates target genes containing CArG elements to promote transcriptional expression of target genes. Finally, HSCs are activated and express a large amount of α-SMA and extracellular matrix components such as type Ⅰ, Ⅲ, Ⅳ collagen.

It can be seen from the above description that there are interactions and crosstalk between the above three mechanotransduction signal pathways in the regulation of HF. Figure 2 briefly summarizes the regulation process of the Hippo-YAP/TAZ signaling pathway, RhoA-ROCK signaling pathway, and MRTF-A signaling pathway on HF.

### 5.4 FAK signaling pathway

Focal adhesion kinase (FAK) is a non-receptor protein tyrosine kinase in the cytoplasm that plays an important role in cellular behavior (adhesion, migration, proliferation, and angiogenesis) and is considered an important component involved in early events in cell-ECM interactions (Yu et al., 2022). FAK is also a key component of mechanotransduction, participating in multiple core signaling pathways and sensing mechanistic cues of extracellular input to regulate cellular states (Wan et al., 2018; Urciuoli and Peruzzi, 2020). In addition to the central kinase domain, FAK has an N-terminal FERM domain and a C-terminal focal adhesion targeting (FAT) domain (Tapial Martínez et al., 2020). The N-terminus is responsible for targeting FAK to the integrin β subunit or growth factor receptor aggregation site, regulating interactions with other potential activator proteins, while the C-terminus contains several protein-protein interaction sites and FAT, which binds to integrin-associated proteins and targets FAK to the focal adhesion complex, which is key to FAK-dependent cell migration (Zhao et al., 2017; Le Coq et al., 2022).

Extracellular mechanical forces stimulate integrin β1 to activate the FAK signaling pathway. Activated FAK promotes cell migration and invasion and mediates the activation of HSCs, differentiation into myofibroblasts, and inhibition of apoptosis (Horowitz et al., 2012; Zhao et al., 2017). A study of breast cancer cells mentioned that rigid ECM can directly stimulate integrin β1 and FAK, accelerate FAs maturation, and induce activation of RhoA/ROCK1/p-MLC and RhoA/ROCK2/p-cofilin signaling cascades, while ROCK1 phosphorylated myosin can regulate light chain and promote traction force generation, and ROCK2 phosphorylated cofilin inhibits F-actin depolymerization to remodel cytoskeleton and finally regulate cell migration (Peng et al., 2019). In liver tissue, activated FAK promotes the migration of HSCs stimulated by platelet-derived growth factor-BB (PDGF-BB) stimulation through small Rho GTPases (Rac and Rho) to accelerate the progression of fibrosis. PDGF is an active factor in a variety of cell migrations and plays an important role in HSCs migration (Heldin and Westermark, 1999). Tyr397 is a key site for FAK-dependent signaling, and its autophosphorylation is a trigger for cellular mechanotransduction. More in detail, FAK can be directly activated by mechanical forces applied at FAs, by inducing conformational changes in FAK structure and subsequent triggering of focal adhesion-mediated signals (Bauer et al., 2019). The phosphorylation level of FAK-Tyr397 was found to be significantly increased in the fibrotic liver tissue of carbon tetrachloride-treated mice, and the expression levels of α-SMA and collagen were also significantly increased, indicating that increased FAK activation (through phosphorylation of Y397 of FAK) was the key to HSCs activation and ECM generation (Zhao et al., 2017). In addition, overexpression of FRNK or blockade of growth factor receptor signaling could inhibit the activity of FAK and lead to apoptosis, and treatment with TGF-β1 and FAK inhibitors confirmed that FAK played an important role in the apoptosis inhibition of TGF-β1-activated HSCs (Parsons, 2003; Zhao et al., 2017). From the above results, in liver tissue, the change of ECM mechanics can activate the FAK signaling pathway, promote the activation of HSCs and myofibroblast differentiation, lead to the increase of ECM proteins (such as α-SMA and collagens), increase the migration of HSCs and resist apoptosis, thus promoting the progression of HF.

### 5.5 Wnt/β-catenin signaling pathway

The Wnt/β-Catenin signaling pathway is one of the highly conserved signaling pathways activated in the evolution of species and can regulate cell proliferation and cell polarity, control cell fate, maintain homeostasis in embryos, and affect the growth and development of tissues and organs (Logan and Nusse, 2004). However, abnormal activation of this pathway may lead to disease (Logan and Nusse, 2004; Ota et al., 2016). The activation of the Wnt/β-Catenin signaling pathway depends on the binding of its ligand to the cell surface curl receptor and low-density lipoprotein receptor-related protein 5/6 (LRP5/6) receptor and can also form an integrin-adhesion complex (IAC) activation pathway by integrin sensing mechanical signals, whereas the final effect of the mechanically signal-activated Wnt/β-Catenin signaling pathway depends on the type of cell (Astudillo, 2020).

The stiff ECM can enhance the expression of each member of the Wnt/β-Catenin signaling pathway in bone marrow mesenchymal stem cells and primary chondrocytes, but the activation of β-Catenin by mechanical signals does not depend on Wnt signaling, but through the activation of integrins forming IAC or activating the FAK pathway, the β-Catenin accumulated in the cytoplasm then translocates to the nucleus to bind to the Wnt1 promoter region, promote gene transcription, and finally form positive feedback to the Wnt/β-Catenin signaling pathway; that is, the activation of β-Catenin by integrins leads to the increased expression of Wnt1, which in turn activates the Wnt/β-Catenin signaling pathway (Du et al., 2016). Moreover, activation of the Wnt/β-Catenin signaling pathway by mechanical signals can regulate the secretion of Wnt ligands, and the expression of several Wnt ligands is reduced when human embryonic stem cells (HESCs) are cultured in higher stiffness substrates, while in bone marrow mesenchymal stem cells (BMSCs), increased substrate stiffness leads to increased Wnt expression and induces activation of the Wnt/β-Catenin signaling pathway (Du et al., 2016; Przybyla et al., 2016). When the Wnt ligand binds to the LRP5/6 receptor, the domain of LRP5/6 in the cytoplasm is phosphorylated, the β-Catenin destruction complex is shifted to the cytoplasmic side of the plasma membrane, β-Catenin cannot be degraded by the destruction complex, and subsequently, the stable β-Catenin is transferred to the nucleus to bind to the Tcf/Lef transcription factor and finally activate the transcription of the target gene (Li et al., 2012; Astudillo, 2020). Thus, there may be a synergy between the integrin approach and the Wnt ligand approach during the activation of the Wnt/β-Catenin signaling pathway by mechanical signaling.

Previous studies (Li W. et al., 2014; Ge et al., 2014) have shown that abnormal activation of β-Catenin plays a key role in HF, and they have found that the more severe the degree of HF, the higher the expression of β-Catenin (compared with normal liver tissue). Collagen secretion and proliferation of HSCs were also inhibited when β-Catenin activity was inhibited, whereas β-Catenin overexpression was observed in the activated HSC-T6 cell membrane and cytoplasm and even in the nucleus. The expression of α-SMA in HSC-T6 cells was decreased after blocking the Wnt/β-Catenin signaling pathway, along with a significant decrease in collagen secretion, suggesting that this pathway is activated in activated HSCs. At present, the mechanism of the Wnt/β-Catenin pathway involved in HF is not completely clear, but it plays an important role in the activation of HSCs and the expression of collagen, which may become a potential therapeutic target for HF.

### 5.6 PIEZO1 ion channel

Piezo1 and Piezo2 are members of a novel class of mechano-activated cation channels (MACs) that mediate cellular biological behaviors, are expressed differently in different cell types, and regulate physiological functions differently (Coste et al., 2010; Wu J. et al., 2017). Piezo1 can interact with the extracellular mechanical microenvironment, while Piezo2 plays an important role in the neurosensory system (Li J. et al., 2014; Ranade et al., 2014; Romac et al., 2018; Wang et al., 2020). In a study (He et al., 2021) on the promotion of cicatrix formation through the activation of the Piezo ion channel by mechanical stretching, we found that the expression of Piezo1 in human dermal fibroblasts (HDFs) exposed to different tensile strengths was in direct proportion to the tensile strength, and the expression of Piezo1 was related to the duration of tension and increased with time. Furthermore, the study found that Piezo1 was involved in mechanically induced HDFs proliferation and motion by flow cytometry assay and Transwell migration assay, and Western blot evaluation found that the α-SMA protein of HDFs under cyclic mechanical stretch (CMS) culture was overexpressed, indicating that the Pizeo1 ion channel can mediate the proliferation, migration, and activation of fibroblasts after mechanical signal activation. In a study of zebrafish cardiac outflow tract valve morphogenesis, Piezo was found to regulate YAP localization and expression of Klf-2 and ECM proteins, suggesting that Piezo channels act through the YAP 1 and Klf 2-Notch signaling axes (Duchemin et al., 2019). In LSECs, mechanical stress can be sensed by integrins, and molecular interactions between integrins and Piezo1 then activate the Piezo channel and allow it to bind to the Notch1 receptor, leading to the expression of downstream transcription factors Hes1 and Hey1, ultimately upregulating Chemokine (C-X-C motif) ligand 1 (CXCL1) production (Hilscher et al., 2019). CXCL1 acts as a neutrophil chemoattractant and induces hepatic sinusoidal thrombosis, portal hypertension, and fibrosis (Hilscher et al., 2019).

At present, the research on Piezo1 ion channel mechanotransduction mainly focuses on the tumor, skin scars, and atherosclerosis (Li et al., 2015; Zhang et al., 2018; Pan et al., 2022), and related research in liver disease is still lacking. Piezo1 is a mechanically sensitive ion channel and is widely expressed in various organ tissues. Piezo1 is a mechanosensitive ion channel, which is widely expressed in various organs and tissues. It has been found that mechanical stretch increases the expression of CXCL1 in LSECs by activating Piezo channels, recruits neutrophils, generates sinusoidal microvessels and promotes portal hypertension and fibrosis. Although the detailed mechanism is not clear, Piezo ion channels may also play a key role in the process of HF, which is a good inspiration.

### 5.7 Other potential mechanotransduction pathways

It has been found that SDC-4 can participate in mechanotransduction, activate mitogen-activated protein kinase (MAPK) and protein kinase C-α (PKC-α) signals in response to directly applied mechanical tension (Bellin et al., 2009; Huang et al., 2015), and can also form a complex with integrin-α5β1, synergistically bind Thy-1 (CD90), form a unique dynamic capture bond, and strengthen under the action of force to mediate adhesion signals (Fiore et al., 2014). SDC-4 has been identified as a mechano-force sensor within cells that regulates the cell’s response to local tension by coordinating mechanochemical signals involving the activation of two other receptors: epidermal growth factor receptor (EGFR) and β1 integrin. Tension on SDC-4 induces cell-wide activation of the kindlin-2/β1 integrin/RhoA axis in a phosphoinositide 3-kinase (PI3K)-dependent manner and initiates conformational changes in the cytoplasmic domain, whose variable region is essential for mechanical adaptation to force, facilitating assembly of the SDC-4/a-actin/F-actin molecular scaffold at the bead adhesion (Chronopoulos et al., 2020). In addition, the data from this study also indicate that SDC-4-mediated tension is required for YAP activation at the cell-ECM interface and that SDC-4 mechanical signals are chemically and mechanically propagated throughout the cell through the diffusion of PIP3 lipid second messengers and mechanically transmitted through the SDC-4-α-actin-F-actin molecular scaffold by stretching the cytoskeleton. Thus, this mechanotransduction pathway of SDC-4 is important for mechanobiology and cell-ECM interaction, but its specific mechanism in hepatocytes and HF is not clear. In the future, the SDC-4 mechanotransduction pathway may be a meaningful direction for the research of mechanotransduction pathways in HF.

The transcriptional coactivator YAP/TAZ, also known as a convergent effector of the Hippo pathway, is widely recognized for its novel role as a nuclear mechanosensor in organ homeostasis and cancer processes. Agrin is known for its ability to aggregate acetylcholine receptors (AChR) at the neuromuscular junction, can act as an ECM sensor, and has functions to stabilize FAs and promote HCC (Chakraborty et al., 2015), and acts as an ECM signal that can be recognized by the integrin/Lrp4/MuSK receptors, which is necessary and sufficient to maintain YAP activity in response to mechanical changes (Chakraborty et al., 2017; Xiong and Mei, 2017). An integrated review from Chakraborty and Hong (2018) suggested that Agrin could be linked to liver cancer through the Hippo Pathway, mainly as: 1) Agrin can activate and stabilize YAP: Elevated agrin levels enhance hepatic ECM stiffness and remodeling by activating YAP, while soluble agrin binds to Lrp4/MuSK and integrins in HCC cells and stabilizes FAs by activating the Foca adhesion kinase-Integrin linked kinase-p21-Activated kinase (FAK-ILK-PAK1) axis; 2) Agrin stimulates the integrin-fak-ILK-PAK 1 mechanistic signaling pathway and antagonizes the Hippo pathway by inhibiting the core Hippo components Merlin and LATS1/2 kinases, thereby activating YAP/TAZ-mediated gene transcription. In addition, agrin-mediated mechanical signaling enhances cell contractility and imparts matrix stiffness by “mechanically activating” YAP-mediated transcription. The sequence of development of hepatitis, HF, and cirrhosis (advanced HF), liver cancer is considered to be the classic trilogy of liver cancer, and although the role of Agrin in activating YAP/TAZ or mediating cell/matrix stiffness is less studied, understanding the agrin-YAP mechanotransduction pathway may help develop therapeutic strategies for diseases including HF, liver cancer.

Osteopontin (OPN), also known as secreted phosphoprotein 1 (SPP1), is a highly negatively charged cytokine-like protein that regulates ECM turnover and remodeling (Bailey et al., 2020). After the liver injury, hepatocytes and inflammatory cells can secrete OPN, but HSCs produce but do not secrete OPN (Song Z. et al., 2021). Serum OPN levels increase progressively with the fibrosis stage in chronic liver disease (Sobhy et al., 2019) and are also considered a marker of the severity of cirrhosis due to alcoholic cirrhosis and NASH (Khajehahmadi et al., 2020). In the process of HF, OPN can activate HSCs through a TGF-β-dependent mechanism and induce type I collagen deposition, which is induced by the participation of αvβ3 integrin and the activation of the PI3K-pAkt-NFκB signaling pathway, but this is a biochemical signal stimulation process. However, in an *in vitro* culture system using adjustable matrix stiffness, OPN expression was significantly upregulated in HCC cells with increased matrix stiffness, accompanied by phosphorylation of GSK-3β and expression of nuclear β-catenin, and analysis of integrin β1 knockdown further suggested that high matrix stiffness upregulated OPN expression in HCC cells by activating integrin β1/GSK-3β/β-catenin signaling pathway (You et al., 2015). In a controlled design, liver stiffness, TAZ expression, hepatic OPN gene expression, and serum OPN protein levels were significantly increased in patients with cirrhosis compared with normal liver tissue, and liver stiffness was significantly correlated with nuclear TAZ and OPN expression (Khajehahmadi et al., 2020). The specific correlation between matrix stiffness as a mechanical property, TAZ and β-catenin as potential mechanical force sensors, and OPN as a matrix cytokine suggests that ECM mechanical properties may affect hepatic OPN expression through mechanical transduction pathways, but the specific mechanism needs to be further confirmed. In addition, whether the upregulated OPN in the liver can further feedback the mechanical properties of ECM (such as ECM stiffness and remodeling) through mechanical transduction pathways to affect the progression of liver disease has not been studied.

## 6 Therapeutic targets: Inhibition or reversal of hepatic fibrosis

Fibrosis is a complex process involving a variety of cell types and a wide range of soluble factors (chemokines, cytokines, and non-peptide mediators), and HF is an inevitable process of liver cirrhosis and the main risk factor for the development of HCC (Weiskirchen et al., 2019). Matrix stiffness is the cause and result of HF. In the early stage of fibrosis, HSCs are activated to become myofibroblasts through the mechanical signal transduction pathway, and a large amount of ECM is generated to promote the stiffness of the matrix. Finally, the stiffened matrix further stimulates HSCs to form a vicious cycle. At present, the research focus is on the targeted therapy of HF, which may be the key to breaking the circulation and inhibiting or reversing HF.

### 6.1 Regulate extracellular matrix homeostasis and reduce extracellular matrix stiffness

Liver ECM stiffness is the main extracellular force that generates mechanical forces, and treatment of ECM stiffness can effectively reduce matrix stiffness and even inhibit or reverse HF. Changes in ECM components often cause an imbalance in matrix generation and degradation. From this perspective, we can search for the influencing factors of the imbalance and intervene with them to achieve the purpose of reducing the stiffness of liver ECM, thereby inhibiting the activation of HSCs and completing the remission or reversal of fibrosis.

In the normal liver, MMPs and TIMPs are in dynamic balance, so ECM can be expressed and degraded normally. The biochemical basis of HF is the metabolic imbalance of the ECM. If the production of ECM is greater than degradation, HF will form and develop; otherwise, HF will resolve and reverse. When HF occurs, TIMP-1 inhibits the activity of MMPs, which prevents collagen degradation and promotes HF. Therefore, the degradation of collagen (I, III) is the key to the reversal and regression of HF. In a study of the effect of the farnesoid X receptor agonist obeticholic acid (OCA) combined with the dipeptide kinase-4 inhibitor sitagliptin on a rat model of HF(Shimozato et al., 2019), the combined use of the two can inhibit the formation of HF and the proliferation of hepatocytes and directly inhibit activated HSCs, significantly reduce the gene expression of TIMP-1 and increase the expression of MMP-2, while TIMP-1 and MMP-2 changes in expression can affect the balance of ECM components, making ECM production less than degradation, and inhibit the progression of HF to a certain extent. Therefore, OCA + sitagliptin may be a promising therapeutic strategy for the treatment of HF.

LOX has the effect of increasing tissue stiffness and resisting ECM degradation, which is achieved by collagens crosslinking to resist the degradation of MMPs, thereby promoting excessive ECM accumulation in the liver and limiting the reversal of the process. In normal physiological processes, LOX mainly maintains the tensile strength and structural integrity of most tissues, and its protein expression is strictly regulated (Barker et al., 2012). In chronic liver disease, the expression of LOX enzymes is upregulated (especially in cirrhosis), the main source of which is activated HSCs(Perepelyuk et al., 2013). In a mouse model of CCl4-induced HF, the use of beta-aminopropionitrile (BAPN, an irreversible LOX inhibitor) was able to inhibit collagen cross-linking and hepatic scar tissue structure, halt the progression of fibrosis and make it easier to reverse fibrosis (Liu et al., 2016). Resveratrol has the effect of reducing collagens fiber bundles, LOX protein levels, and visceral hydroxyproline content in a Wistar rat model of HF and inhibiting fibrosis, and it can better inhibit HF when used together with BAPN(Mohseni et al., 2019).

### 6.2 Reduction of activated HSCs and inhibition of HSCs response to matrix stiffness

In addition to the fact that cells are stimulated by and respond to their external signaling molecules, biomechanics can also be the reason for cell behavior changes (Discher et al., 2005). HSCs and their activation products, myofibroblasts, can be stimulated by the sclerotic extracellular environment to overproduce ECM and related proteins, further promoting the stiffness of the extracellular environment. Activated HSCs increase the expression of α-SMA and the production of hydroxyproline, where α-SMA is an activation marker of HSCs and can be used for detection. Therefore, inducing apoptosis of HSCs or myofibroblasts or blocking and inhibiting their response to mechanical signals is a potential therapeutic approach.

The Rho-ROCK signaling pathway is one of the mechanical signaling pathways of HSCs perception and effect, which participates in the activation of HSCs, and inhibition of this pathway can inhibit the activation of HSCs and improve HF. P160ROCK (Y27632) is a pyridine derivative that can specifically inhibit the ROCK protein kinase family (Uehata et al., 1997). In the experimental hepatic fibrosis model induced by dimethylnitrosamine (DMN), the incidence of HF in rats orally administered Y27632 was significantly reduced, the proliferation and diffusion of HSCs were continuously reduced, the expression of hydroxyproline and α-SMA were downregulated, fiber assembly was significantly reduced, and the transcription level of type Ⅰ collagen mRNA was significantly inhibited (Tada et al., 2001).

YAP is a transcriptional coactivator and one of the key downstream proteins in the Hippo signaling pathway, but it is also cross-linked with the Rho signaling pathway and is also a potential therapeutic target. Influenced by the positive feedback of matrix stiffening, YAP is activated and subsequently translocated to the nucleus to direct the expression of genes such as cell proliferation, apoptosis, and motility (Lachowski et al., 2018). Studies (Yu et al., 2019) have shown that the injection of CCl4 into mice can lead to the upregulation of YAP expression in HF tissues and restore it to the normal level after 6 weeks of stopping the injection, and TGF-β1-treated HSCs also upregulate the expression of YAP. However, after silencing of YAP, we found that the apoptosis of HSCs was enhanced, and the activation and proliferation of HSCs were decreased but apoptosis was increased after treatment with verteporfin (VP, YAP-TEAD complex inhibitor). YAP plays a central role in the activation and proliferation of HSCs, and reducing and inhibiting the expression of YAP helps to reverse HF.

The TGF-β1/Smads and PI3K/Akt pathways are involved in HSCs activation and autophagy. TGF-β1 is secreted by activated HSCs and is one of the main factors that induce collagen production and lead to matrix deposition and can upregulate TIMPs(Xu et al., 2012). Quercetin (QE) is a natural flavonoid that has good safety and bioavailability, as well as anti-inflammatory and antitumor activities (Harwood et al., 2007; Jagtap et al., 2009). Wu et al. established a mouse model of HF by bile duct ligation (BDL) and CCl4 injection to study the effect of QE on HF and found that QE could reduce fibrosis in both modeling methods. After QE treatment, ECM formation was inhibited, MMP-9 and TIMP-1 were regulated, and QE inhibited the TGF-β1/Smads signaling pathway and activated the PI3K/Akt signaling pathway, thereby inhibiting HSCs activation, reducing HSCs autophagy, and inhibiting HF(Wu L. et al., 2017).

Biological and biomechanical studies have shown that the overexpression of FAK and the increase of FAK activity play an important role in the occurrence and development of HF. FAs are discrete multiprotein complexes formed at internal sites of the cell membrane by cells attached to a stiff matrix, are a major component of the interaction between ECM and cells, and are responsible for sensing and translating mechanical stimuli generated by ECM to the cellular cytoskeleton, whereas FA assembly/disassembly and integrin-mediated mechanotransduction can respond to ECM stiffness (Urciuoli and Peruzzi, 2020). FAK phosphorylation is the first event in response to integrin-mediated cell adhesion (Kornberg et al., 1991), and inhibition of FAK phosphorylation may reduce ECM stiffness (e.g., reduce α-SMA and collagens expression) and thus prevent HF progression. In the study of Xing et al. (2021), it was found that the increase of ECM stiffness can activate the integrin β5-FAK-MEK1/2-ERK1/2 pathway to promote the expression of lysyl oxidase-like 2 (LOXL2). Although this is a potential mechanism for ECM rigidification to induce the formation of a premetastatic niche in HCC, it also shows that the increase of ECM stiffness can promote the phosphorylation of FAK by stimulating integrin β5. In a study of HF in mice treated with carbon tetrachloride, TGF-β1 induced FAK activation in a time-and dose-dependent manner, and FAK activation was associated with increased expression of α-SMA and collagens in fibrotic liver tissue, while inhibition of FAK activation (Y397 phosphorylation of FAK) with FAK inhibitors (PF 562271) blocked α-SMA and collagen expression in TGF-β1-treated HSCs and induced apoptotic signaling, inhibiting the formation of stress fibers, thereby attenuating HF in mice (Xing et al., 2021). In the study of Lv et al. (2018), artesunate strongly inhibited the proliferation of LX-2 cells in a dose and time-dependent manner, inhibited the proliferation and activation of HSCs by inhibiting the phosphorylation level of FAK, attenuated the expression of genes encoding α-SMA and type I collagen, and promoted the apoptosis of HSCs.

## 7 Conclusion

Chronic liver injury can induce activation of HSCs and secretion of excessive ECM protein through inflammatory response, destroy ECM homeostasis, lead to abnormal deposition of ECM, increase matrix stiffness, and promote the occurrence and development of HF. The stiff ECM alters the local cell tension and regulates the biological behavior of HSCs through mechanotransduction pathways. At present, mechanotransduction pathways have been found to have potential and application prospects in the diagnosis and treatment of HF, but our understanding of mechanosensing and intercellular mechanotransduction mechanisms is still limited. In addition, in the course of future studies, we also need to take into account that mechanotransduction and biochemical signaling in the liver interact and crosstalk to produce synergistic effects on HF. A better understanding of how hepatocytes (e.g., HSCs, hepatic macrophages) sense matrix stiffness and translate matrix mechanical cues into cellular responses, as well as the interactions and cross-talk between mechanotransduction and biochemical signaling in the liver, could provide us with more opportunities to develop targeted drugs or inhibitors that target altered ECM mechanisms in HF. More importantly, in future studies, we also need to consider combination strategies and drugs targeting biochemical and mechanical signals to improve clinical outcomes in patients with HF. At present, although some *in vitro* or animal models for matrix stiffness intervention have achieved initial results, a lot of work is needed to further verify their efficacy in the future.
