# Supplementary material for: Mechanical homeostasis imbalance in hepatic stellate cells activation and hepatic fibrosis
Source: Front Mol Biosci. 2023 Apr 20;10:1183808. doi: 10.3389/fmolb.2023.1183808 (PMC10157180; doi:10.3389/fmolb.2023.1183808)
Supplement: Supplementary file 1 [file Table1.DOCX]

Table1. The differences between myofibroblasts versus quiescent HSCs that cause ECM imbalance and hepatic fibrosis.

| Quiescent HSCs | Myofibroblasts |
| --- | --- |
| Cell bodies ovoid or irregularly shaped and often projecting several stellate projections | Cell bodies are larger and the stellate projections are stretched and thinned |
| Rich in retinoid droplets | Retinoid loss |
| ECM homeostasis | α-SMA and collagen-I abundant expression |
| Non-proliferative | Proliferation |
| Expression of small amounts of TGF-β, PDGF, IGF and other cytokines | Accumulation of ECM proteins |
|  | Contractility and chemotaxis |
|  | Increased cross-linking and promoting Fibrogenesis |
|  | Altered matrix degradation |
|  | Involved in inflflammatory and mechanical signaling |
